# 23154 Electrical stimulation of hippocampus and amygdala produces multiple distinct responses in human ventral temporal cortex

**DOI:** 10.1017/cts.2021.639

**Published:** 2021-03-31

**Authors:** Harvey Huang, Nicholas M. Gregg, Gabriela Ojeda, Benjamin H. Brinkmann, Brian N. Lundstrom, Gregory A. Worrell, Kai J. Miller, Dora Hermes

**Affiliations:** Mayo Clinic

## Abstract

ABSTRACT IMPACT: This study characterizes interactions between human limbic circuitry and ventral temporal cortex using single pulse electrical stimulation, which may inform emerging stimulation therapies for epilepsy. OBJECTIVES/GOALS: The goal of electrical brain stimulation treatment is to modulate brain network function. However, stimulation inputs to different brain sites alter the network in a variety of ways. This study examines that variability by characterizing responses in a target region while stimulating multiple other brain sites. METHODS/STUDY POPULATION: We measured voltages in intracranial EEG in 6 patients who had electrodes implanted for epilepsy monitoring. We stimulated pairs of electrodes at multiple sites in the brain with a single pulse every 5 to 7 s and measured the resulting corticocortical evoked potential (CCEP) responses in the ventral temporal cortex (VTC). Using a novel clustering method, we uncovered sets of distinct canonical response shapes from the 20 to 500 ms post-stimulation period. This allowed us to group stimulation sites that evoked similar responses. We then related each group to high frequency, broadband, changes in spectral power as a reflection of local neuronal activity. RESULTS/ANTICIPATED RESULTS: We found that the VTC receives strong inputs specifically from the amygdala and hippocampus, both in terms of amplitude and broadband spectral power change. However, inputs from the hippocampus produced a different canonical shape than those from the amygdala. We also observed that VTC responses to inputs from the insula clustered in shape with those from the amygdala. These clustering patterns were consistent across subjects, although the actual shapes of the clusters showed variability. We further observed that some shapes were more associated with increases in overall neuronal activity than others, as reflected by broadband spectral power change. DISCUSSION/SIGNIFICANCE OF FINDINGS: Stimulation of connected sites may drive excitability at the target region in ways that are described by sets of full-time-course responses. By capturing their shapes, we can begin to decipher canonical input types at the circuit level. This approach might identify how stimulation inputs can be tailored to therapy while mitigating adverse effects.

